# Epidemiology of uveitis (2013–2015) and changes in the patterns of uveitis (2004–2015) in the central Tokyo area: a retrospective study

**DOI:** 10.1186/s12886-018-0871-6

**Published:** 2018-08-02

**Authors:** Shintaro Shirahama, Toshikatsu Kaburaki, Hisae Nakahara, Rie Tanaka, Mitsuko Takamoto, Yujiro Fujino, Hidetoshi Kawashima, Makoto Aihara

**Affiliations:** 1 Department of Ophthalmology, University of Tokyo Graduate School of Medicine, 7-3-1 Hongo, Bunkyo-ku, Tokyo, 113-8655 Japan; 2 Department of Ophthalmology, Saitama Red Cross Hospital, 1-5 Shintoshin, Chuo-ku, Saitama-shi, Saitama, 330-8553 Japan; 3 Department of Ophthalmology, Tokyo Shinjuku Medical Center, 5-1 Tsukudo-cho, Shinjuku-ku, Tokyo, 162-8543 Japan; 4 Department of Ophthalmology, Jichi Medical University, 3311-1 Yakushiji, Shimotsuke-City, Tochigi, Japan

**Keywords:** Diagnosis, Epidemiology, Japan, Trend, Uveitis

## Abstract

**Background:**

The distribution of uveitis varies with genetic, ethnic, geographic, environmental, and lifestyle factors. Epidemiological information about the patterns of uveitis is useful when an ophthalmologist considers the diagnosis of uveitis. Therefore, it is important to identify the causes of uveitis over the years in different regions. The purposes of this study were to characterize the uveitis patients who first arrived at the University of Tokyo Hospital in 2013–2015, and to analyze the changes in the patterns of uveitis from 2004 to 2012 to 2013–2015.

**Methods:**

We retrospectively identified 750 newly arrived patients with uveitis who visited the Uveitis Clinic in the University of Tokyo Hospital between January 2013 and December 2015, using clinical records. We extracted data on patient age, sex, diagnosis, anatomic location of inflammation, laboratory test results of blood and urine, and chest X-ray and fluorescein fundus angiography findings for each patient. In addition, we compared these data with those from 2004 to 2012 to analyze the changes in the patterns of uveitis.

**Results:**

A definite diagnosis was established in 445 patients (59.3%). The most common diagnoses were herpetic iridocyclitis (7.5%), sarcoidosis (6.1%), Behçet’s disease (4.4%), Vogt–Koyanagi–Harada disease (4.1%), and intraocular lymphoma (4.1%). The most frequent unclassified type of uveitis was suspected sarcoidosis (22.3%). Analysis of the changes in the patterns of uveitis in the central Tokyo area from 2004 to 2012 to 2013–2015 revealed notable increasing trends of herpetic iridocyclitis and intraocular lymphoma, and increasing trends of bacterial endophthalmitis, fungal endophthalmitis, and juvenile chronic iridocyclitis. In contrast, the frequency of sarcoidosis, Behçet’s disease, and Vogt–Koyanagi–Harada disease decreased.

**Conclusions:**

The patterns of uveitis changed considerably from 2004 to 2012 to 2013–2015. Continuous investigations about the epidemiology of uveitis are needed to diagnose uveitis more accurately.

## Background

Uveitis is a leading cause of visual blindness in developed countries [1]. Many studies have reported the patterns of uveitis in different countries and ethnicities [2–7]. The distribution of the types and etiologies of uveitis is influenced by genetic, ethnic, geographic, environmental, and lifestyle factors [8]. As a result, the patterns of uveitis vary greatly according to the population and the time of research. For example, one report from Taiwan presented data demonstrating that the incidences of herpetic anterior uveitis, acute retinal necrosis, and cytomegalovirus (CMV) retinitis have increased, while those of toxoplasmosis and tuberculosis have decreased [9], compared with findings of a previous study [10]. Therefore, it is important to analyze the epidemiology of this disease in various regions over time.

In recent years, highly advanced diagnostic methods for uveitis, including optical coherent tomography and polymerase chain reaction (PCR) analysis for infectious agents using aqueous samples, have been developed. Consequently, the number of definite diagnoses has been gradually increasing [2]. However, a definite diagnosis can still not be reached in approximately 30–40% of patients newly diagnosed with uveitis [2, 4, 5, 11].

Region-specific information about the patterns of uveitis is helpful when the clinicians consider the diagnosis for newly arrived patients. There are many reports on this topic from both Japan and other countries [3–7, 12]. Previously, we have reported the patterns of newly arrived patients with uveitis at the University of Tokyo Hospital between 2004 and 2012 [2, 11]. In this institution, retrospective analysis of newly arrived patients with uveitis have been conducted over the past 50 years [2]. Therefore, we consider that the data from our hospital are representative of the changing patterns of uveitis in Japan.

In the current study, we investigated the records of patients who visited the University of Tokyo Hospital during 2013–2015 and compared the results to those of our previous studies (2004–2012) [2, 11].

## Methods

We retrospectively investigated the clinical records of 750 newly arrived patients with uveitis (363 men, 387 women) who first visited the Uveitis Clinic of the University of Tokyo Hospital (a tertiary referral center located in central Tokyo) between January 2013 and December 2015. Patients with uveitis who had first visited before 2012 due to uveitis were excluded from the study. Conversely, patients who had first visited our clinic before 2012 for other reasons and then presented with uveitis during the study period were included in this study.

We collected clinicodemographic data, including age, sex, diagnosis, anatomic location of inflammation, laboratory test results of blood and urine, and chest X-ray and fluorescein fundus angiography findings from the patients’ clinical records. The ethics committee of the University of Tokyo Hospital allowed us to collect clinical data for this retrospective study.

We adopted the uveitis classification method used in a nationwide investigation of uveitis conducted in 2009 in Japan [13]. The anatomic diagnosis was evaluated according to the classification of the Standardization of Uveitis (SUN) Working Group, as anterior uveitis, intermediate uveitis, posterior uveitis, or panuveitis [14].

All patients with uveitis underwent blood tests (peripheral blood count, erythrocyte sedimentation rate, serum angiotensin-converting enzyme, glucose, rheumatoid factor, immunoglobulin [Ig] A, IgG, IgM, antinuclear antibody (double-stranded deoxyribonucleic acid [DNA] antibody and single-stranded DNA antibody), calcium, blood urea nitrogen, creatinine, creatine kinase, rapid plasma regain, *Treponema pallidum* latex agglutination, anti-human T-cell lymphotropic virus type I [HTLV-1] antibody, C-reactive protein, globulin fraction, IgM and IgG of toxoplasma, herpes simplex virus [HSV], varicella zoster virus [VZV], and CMV), urine tests, chest X-ray examination, and the Mantoux reaction test at the initial presentation. Additionally, PCR tests, β-d-glucan, HLA, interferon gamma release assays, diagnostic vitrectomy, and fluorescein angiography were performed when specific uveitis diseases were suspected; those examinations might be useful for judging the diagnosis. Quantitative PCR using aqueous humor and blood cultures was conducted when infectious uveitis was suspected; serum β-d-glucan testing (for suspected fungal endophthalmitis) and human leukocyte antigen (HLA) typing (for suspected acute anterior uveitis or Behçet’s disease) were also performed. Interferon-gamma release assays were conducted when the Mantoux reaction test was strongly positive. Moreover, vitreous fluid examinations were conducted when intraocular lymphoma was suspected. If inflammation was suspected in retina and/or optic disc, fluorescein angiography was performed if the patient consented to the procedure. Information obtained from fluorescein angiography was used to determine the anatomical location of uveitis and the presence or absence of retinal vasculitis and/or chorioretinitis for consideration in differential diagnosis of uveitis.

Behçet’s disease was diagnosed based on criteria established by the Behçet’s Disease Research Committee of Japan [15]. For sarcoidosis, we used the criteria established by the Japanese Society of Sarcoidosis and Other Granulomatous Disorders [16, 17]. For Vogt–Koyanagi–Harada disease (VKH), we adopted previously reported criteria [18]. For herpetic iritis [19], patients with active skin lesions associated with ophthalmic herpes zoster (i.e., the vesicular rash involving the periocular skin, such as the eyelids, medial canthal area, or the tip of the nose, known as Hutchinson’s sign) were clinically diagnosed and classified as VZV iritis. Patients without a skin lesion were subjected to PCR assays for HSV, VZV, and CMV DNA using anterior chamber fluid. The presence of > 100 copies/mL of viral DNA were judged as a positive finding. Patients with negative PCR results for HSV, VZV, and CMV DNA, and good response to anti-herpetic treatment were classified as herpetic iridocyclitis (clinical diagnosis), while those who did not undergo PCR assays were classified as suspected herpetic iritis. The diagnoses of bacterial and fungal endophthalmitis were based on matching of ocular symptoms suggesting bacterial or fungal etiology, the results of laboratory tests (blood culture, serum β-d-glucan), detection of bacterial or fungal DNA in aqueous humor samples by broad-range PCR [20, 21], and a response to antibiotics or antifungal drugs. As for intraocular lymphoma, we diagnosed this disease if at least two of the following four criteria were met: cytology >class 3, interleukin (IL)-10/IL-6 ratio > 1 or IL-10 > 50 pg/mL in the intraocular fluid [22], κ/λ ratio on fluorescence-activated cell sorting analysis, and positive PCR results for immunoglobulin heavy chain (IgH) gene rearrangement [23]. Regarding acute anterior uveitis (AAU), patients with unique symptoms of ankylosing spondylitis, ulcerative colitis, or psoriasis were diagnosed as having systemic disease-associated uveitis. Those with HLA-B27 were diagnosed as AAU, while those without HLA-B27 or with unknown HLA typing were diagnosed as unclassified uveitis. We diagnosed ocular tuberculosis based on a combination of ocular symptoms indicating tuberculous etiology, laboratory tests, and response to anti-tuberculosis therapy. Consequently, we used the diagnostic criteria for presumed ocular tuberculosis [24]. The diagnostic criteria for diabetic iritis in this study were (1) acute severe iridocyclitis in patients with poor glycemic control (HbA1c ≥ 8.0%) and (2) other investigations for systemic disease associated with uveitis were negative [25]. For HTLV-1 [26], acute zonal occult outer retinopathy (AZOOR) [27], and rubella virus-associated uveitis [28], we adopted the criteria described in previous reports [26–28]. Juvenile chronic iridocyclitis was diagnosed by the typical ocular findings and clinical courses, as described in a previous report [29].

Statistical analyses were performed using the χ-squared test in SPSS for Windows, version 14.0 (SPSS Inc., Chicago, IL, USA). We compared the frequency of each disease between 2013 and 2015 with those between 2004 and 2012. A *p* value < 0.05 was considered statistically significant.

## Results

**Fig. 1:**
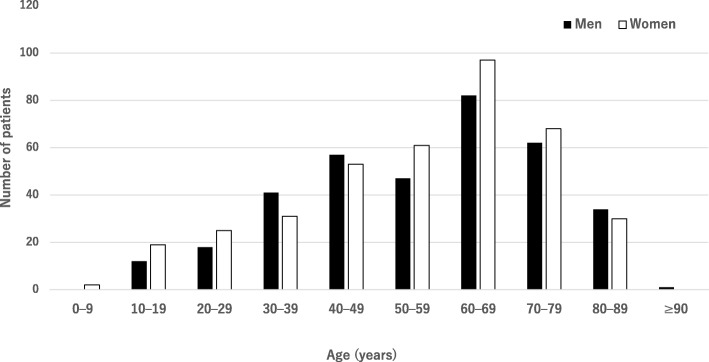
Distribution of 750 patients with uveitis from 2013 to 2015 by age and sex Uveitis was more frequent in the age group of 60–69 years among both men and women

Figure 1 shows the age distribution of the patients with uveitis that visited our institution between 2013 and 2015. The mean age was 56.4 ± 18.5 years (men: 56.9 ± 18.9 years, women: 56.1 ± 18.9 years). The most common age category was 60–69 years for both men and women. Among a total of 750 newly arrived patients with uveitis, 445 patients (59.3%) received a definite diagnosis. Table 1 presents the distribution of patients who were given a definite diagnosis. The three most common diagnoses were herpetic iridocyclitis (HSV, VZV, and CMV) in 56 patients (7.5%), sarcoidosis in 46 (6.1%), and Behçet’s disease in 31 (4.4%).

**Table 1:** Distribution of uveitis among new patients (2013–2015)

Patient diagnosis	No. of patients		Sex	
	No.	%	Male	Female
Herpetic iridocyclitis	56	7.5	43	13
Sarcoidosis	46	6.1	13	33
Behçet’s disease	33	4.4	17	16
Vogt–Koyanagi–Harada disease	31	4.1	12	19
Intraocular lymphoma	31	4.1	21	10
Posner–Schlossman syndrome	25	3.3	15	10
Bacterial endophthalmitis	23	3.1	9	14
Fuchs heterochromic iridocyclitis	20	2.7	11	9
Juvenile chronic iridocyclitis	17	2.3	4	13
Fungal endophthalmitis	16	2.1	11	5
Acute anterior uveitis	14	1.9	8	6
Acute retinal necrosis	13	1.7	5	8
HTLV-1-associated uveitis	13	1.7	5	8
Cytomegalovirus retinitis	10	1.3	6	4
Tuberculosis	9	1.2	6	3
AZOOR	8	1.1	3	5
MEWDS	8	1.1	3	5
Psoriatic uveitis	7	0.9	4	3
Neuroretinitis	6	0.8	3	3
Lens induced uveitis	6	0.8	2	4
Toxoplasma	6	0.8	5	1
IBD	5	0.7	4	1
APMPPE	4	0.5	1	3
Diabetic iridocyclitis	4	0.5	4	0
Systemic lupus erythematosus	4	0.5	0	4
Syphilis	3	0.4	2	1
Rubella	3	0.4	2	1
Ankylosing spondylitis	2	0.3	2	0
Immune reconstitution syndrome	2	0.3	0	2
JIA	2	0.3	0	2
Multifocal choroiditis	2	0.3	1	1
RA	2	0.3	0	2
Sympathetic ophthalmia	2	0.3	1	1
Geographic chorioretinopathy	2	0.3	1	1
Multiple sclerosis	2	0.3	0	2
Sclerouveitis due to scleroderma	2	0.3	0	2
CAR	1	0.1	0	1
PIC	1	0.1	1	0
UAIM	1	0.1	0	1
Iridocyclitis due to nivolumab	1	0.1	0	1
Cat scratch disease	1	0.1	0	1
Polymyalgia rheumatica	1	0.1	0	1
Unclassified uveitis	305	38.4	138	167
Total	750		363	387

*HTLV-1* human T lymphotropic virus type-1, *AZOOR* acute zonal occult outer retinopathy, *MEWDS* multiple evanescent white dot syndrome, *IBD* inflammatory bowel disease, *APMPPE* acute posterior multifocal placoid pigment epitheliopathy, *JIA* juvenile idiopathic arthritis, *RA* rheumatoid arthritis, *CAR* cancer-associated retinopathy, *PIC* punctate inner choroidopathy, *UAIM* unilateral acute idiopathic maculopathy

Patients with herpetic iridocyclitis were divided into five groups by diagnostic method. Of these, CMV DNA positivity was the most common diagnostic method (*n* = 34, 60.7%), followed by skin lesions of herpes zoster ophthalmicus (*n* = 12, 21.4%), VZV DNA positivity (*n* = 5, 8.9%), HSV DNA positivity (*n* = 3, 5.4%), and clinical diagnosis only (*n* = 2, 3.6%) (Table 2).

**Table 2:** Diagnostic methods for herpetic iritis

Diagnostic method	No. of patients (%)
Clinical diagnosis only	2 (3.6)
Skin lesions of herpes zoster ophthalmicus and granulomatous iridocyclitis	12 (21.4)
Herpes simplex virus detection with PCR using aqueous humor	3 (5.4)
Varicella zoster virus detection with PCR using aqueous humor	5 (8.9)
Cytomegalovirus detection with PCR using aqueous humor	34 (60.7)

*PCR* polymerase chain reaction

Table 3 shows the distributions of patients with uveitis according to three age groups (< 20 years, 20–59 years, and ≥ 60 years). In these patients, juvenile chronic iridocyclitis, Behçet’s disease, and herpetic iridocyclitis were the most common diagnoses, respectively. Definite diagnoses were not made for the remaining 305 patients (39.1%). Among these patients, suspected sarcoidosis was the most frequent diagnosis (*n* = 167, 22.3%), which accounted for 54.8% of all suspected diagnoses.

**Table 3:** Frequency of new patients with uveitis (2013–2015) by age

< 20 years (*n* = 31)	No. of patients (%)	20–59 years (*n* = 358)	No. of patients (%)	≥60 years (*n* = 409)	No. of patients (%)
Juvenile chronic iridocyclitis	11 (35.5)	Behçet’s disease	24 (7.1)	Herpetic iridocyclitis	37 (9.7)
Fuchs heterochronic iridocyclitis	3 (9.7)	Herpetic iridocyclitis	19 (5.7)	Sarcoidosis	27 (7.0)
Bechet disease	3 (9.7)	Sarcoidosis	19 (5.7)	Intraocular lymphoma	27 (7.0)
Posner-Schlossman syndrome	2 (6.5)	Vogt-Koyanagi-Harada disease	16 (4.8)	Bacterial endophthalmitis	20 (5.2)
HTLV-1-associated uveitis	1 (3.2)	Posner-Schlossman syndrome	13 (3.9)	Vogt-Koyanagi-Harada disease	14 (3.7)
JIA	1 (3.2)	Acute anterior uveitis	12 (3.6)	Fungal endophthalmitis	12 (3.1)
Cat scratch disease	1 (3.2)	Fuchs heterochromic iridocyclitis	10 (3.0)	HTLV-1-associated uveitis	11 (2.9)
Cytomegalovirus retinitis	1 (3.2)	MEWDS	8 (2.4)	Posner-Schlossman syndrome	10 (2.6)
Vogt-Koyanagi-Harada disease	1 (3.2)	AZOOR	7 (2.1)	Cytomegalovirus retinitis	8 (2.1)
Toxoplasma	1 (3.2)	Juvenile chronic iridocyclitis	6 (1.8)	Fuchs heterochromic iridocyclitis	7 (1.8)
Unknown	6 (19.4)	Acute retinal necrosis	6 (1.8)	Acute retinal necrosis	7 (1.8)
		Tuberculosis	6 (1.8)	Lens induced uveitis	6 (1.6)
		IBD	5 (1.5)	Behçet’s disease	6 (1.6)
		Systemic lupus erythematosus	4 (1.2)	Diabetic iridocyclitis	4 (1.0)
		Psoriatic uveitis	4 (1.2)	Psoriatic uveitis	3 (0.8)
		Intraocular lymphoma	4 (1.2)	Tuberculosis	3 (0.8)
		Neuroretinitis	4 (1.2)	Syphilis	3 (0.8)
		Fungal endophthalmitis	4 (1.2)	APMPPE	3 (0.8)
		Toxoplasma	3 (0.9)	Toxoplasma	2 (0.5)
		Bacterial endophthalmitis	3 (0.9)	Acute anterior uveitis	2 (0.5)
		Rubella virus-associated uveitis	3 (0.9)	Sympathetic ophthalmia	2 (0.5)
		Multiple sclerosis	2 (0.6)	Neuroretinitis	2 (0.5)
		Ankylosing spondylitis	2 (0.6)	AZOOR	1 (0.3)
		Multifocal choroiditis and panuveitis	2 (0.6)	CAR	1 (0.3)
		APMPPE	1 (0.3)	Immune reconstitution syndrome	1 (0.3)
		Cytomegalovirus retinitis	1 (0.3)	Polymyalgia rheumatica	1 (0.3)
		Immune reconstitution syndrome	1 (0.3)	RA	1 (0.3)
		HTLV-1-associated uveitis	1 (0.3)	Sclerouveitis due to scleroderma	1 (0.3)
		JIA	1 (0.3)	Geographic chorioretinopathy	1 (0.3)
		PIC	1 (0.3)	Unknown	160 (41.8)
		RA	1 (0.3)		
		UAIM	1 (0.3)		
		Iridocyclitis due to nivolumab	1 (0.3)		
		Sclerouveitis due to scleroderma	1 (0.3)		
		Geographic chorioretinopathy	1 (0.3)		
		Unknown	139 (41.4)		
Total	31	Total	336	Total	383

*HTLV-1* human T lymphotropic virus type-1, *AZOOR* acute zonal occult outer retinopathy, *MEWDS* multiple evanescent white dot syndrome, *IBD* inflammatory bowel disease, *APMPPE* acute posterior multifocal placoid pigment epitheliopathy, *JIA* juvenile idiopathic arthritis, *RA* rheumatoid arthritis, *CAR* cancer-associated retinopathy, *PIC* punctate inner choroidopathy, *UAIM* unilateral acute idiopathic maculopathy

Table 4 shows the distribution of uveitis categorized by anatomical location (anterior uveitis, intermediate uveitis, posterior uveitis, and panuveitis). In this study, 289 out of 750 patients (38.5%) had anterior uveitis, 12 (1.6%) had intermediate uveitis, 94 (12.5%) had posterior uveitis, and 355 (47.3%) had panuveitis. The distribution of uveitis according to anatomical site did not differ from that in our previous studies [2].

**Table 4:** Shifts in the distribution of anatomic localization of uveitis (2004–2015) [2, 11]

Period	2004–2006^a^	2007–2009^a^	2010–2012^b^	2013–2015
New patients (n)	426	535	695	750
Anterior uveitis	48.8%	48.0%	50.1%	38.5%
Intermediate uveitis	–	–	1.6%	1.6%
Posterior uveitis	9.6%	15.7%	13.4%	12.5%
Panuveitis	41.5%	36.3%	35.0%	47.3%

^a^The classification of the International Ocular Inflammation Society (IOIS) was adopted. Intermediate uveitis is included in the definition of posterior uveitis [27]

^b^The classification of the Standardization of Uveitis Nomenclature (SUN) [14] was adopted

Table 5 shows the shifting numbers and distributions of the uveitis cases diagnosed at the University of Tokyo Hospital over 12 years. Compared with our previous studies since 2004 [2, 11], the present analysis showed that the incidences of herpetic iridocyclitis, intraocular lymphoma, bacterial endophthalmitis, fungal endophthalmitis, and juvenile chronic iridocyclitis have been increasing. Additionally, sarcoidosis, Behçet’s disease, and VKH disease have been decreasing in recent years. However, the increases or decreases of these disease frequencies were not statistically significant (*p* > 0.05 for all).

**Table 5:** Shifts in the number and distribution (%) of diagnosed uveitis cases (2004–2015) [2, 11]

Diagnosis	2004–2006	2007–2009	2010–2012	2013–2015
Herpetic iridocyclitis	20 (4.7)	28 (5.2)	38 (5.5)	56 (7.5)
Sarcoidosis	37 (8.7)	44 (8.2)	56 (8.1)	46 (6.1)
Behçet’s disease	21 (4.9)	26 (4.9)	32 (4.6)	33 (4.4)
Vogt–Koyanagi–Harada disease	27 (6.3)	37 (6.9)	28 (4.0)	31 (4.1)
Intraocular lymphoma	4 (0.9)	13 (2.4)	21 (3.0)	31 (4.1)
Posner–Schlossman syndrome	19 (4.5)	20 (3.7)	25 (3.6)	25 (3.3)
Bacterial endophthalmitis	2 (0.5)	10 (1.9)	13 (1.9)	23 (3.1)
Fuchs heterochromic iridocyclitis	9 (2.1)	10 (1.9)	11 (1.6)	20 (2.7)
Juvenile chronic iridocyclitis	3 (0.7)	7 (1.3)	11 (1.6)	17 (2.3)
Others	90 (21.1)	100 (18.7)	138 (20.0)	163 (21.7)
Unclassified uveitis	147 (34.5)	173 (32.3)	264 (38.0)	305 (40.7)
Total	426	535	695	750

The data are presented as no. of patients (%)

Table 6 reports the distribution of the age of patients at the first visit in this study (2013–2015) and previous studies (2004–2006, 2007–2009, and 2010–2012). The average ages of new patients were 52.0, 51.6, 53.6, and 56.4 years, respectively, indicating that the average age has been gradually increasing.

**Table 6:** Shifts in the numbers of new patients with uveitis according to age (2004–2015) [2, 11]

Age (years)	2004–2006	2007–2009	2010–2012	2013–2015
< 20	11 (2.6)	26 (4.9)	29 (4.2)	31 (3.9)
20–59	251 (58.9)	308 (57.6)	354 (50.9)	336 (44.8)
≥60	164 (38.5)	201 (37.6)	312 (44.9)	383 (51.1)
Total	426	535	695	750

The data are presented as no. of patients (%)

## Discussion

In this study, the most frequent uveitis categories in patients aged < 20, 20–59, and ≥ 60 years were juvenile chronic iridocyclitis, Behçet’s disease, and herpetic iridocyclitis, respectively. A previous study conducted in our hospital (2010–2012) showed that the most frequent types of uveitis in the same age groups were juvenile chronic iridocyclitis, Behçet’s disease, and sarcoidosis, respectively [11]. In comparison with the findings of the previous study, an increase in the incidence of herpetic iridocyclitis in patients aged ≥60 years was observed. A possible reason for this new finding might be the progression of aging in Japan [30]. In fact, the incidence of infections, such as herpes zoster, in the elderly has been shown to be increasing in other countries as well [31].

Compared with the previous studies from our hospital [2, 11], the ratios of herpetic iridocyclitis (7.5%), intraocular lymphoma (4.1%), bacterial endophthalmitis (3.1%), fungal endophthalmitis (2.1%), and juvenile chronic iridocyclitis (2.3%) were increased in this study.

We speculated that the increase of herpetic iridocyclitis patients may be attributed to the recurring use of PCR assays for HSV, VZV, and CMV DNA using anterior chamber fluid. The increase in intraocular lymphoma may be due to two reasons. First, that we passively conducted diagnostic vitrectomy for patients suspected of having intraocular lymphoma. Second, that there has been an increase in the number of patients with primary central nervous system lymphoma [32]. The increases of patients with bacterial and fungal endophthalmitis were considered to be due to advances in the relevant diagnostic methods. Broad-range real-time PCR for bacterial DNA [19] and fungus DNA [20] were employed using both anterior chamber fluid and vitreous fluid. Moreover, the increase of juvenile chronic iridocyclitis might be related to the increasing number of patients aged < 20 years old in our hospital. In Japan, juvenile chronic iridocyclitis was not commonly associated with juvenile idiopathic arthritis [33]. The number of patients with juvenile chronic iridocyclitis without juvenile idiopathic arthritis in this study was also larger than the number of patients with both juvenile chronic iridocyclitis and juvenile idiopathic arthritis.

Notably, the ratios of scleritis, sarcoidosis, Behçet’s disease, and VKH have been gradually decreasing over the past years in our institution. We cannot find adequate reasons for the decreasing frequencies of these diseases.

Another interesting finding of this study is the trend for increasing age of newly arrived patients with uveitis. Compared to the data from 2004 to 2006, the average age of the patients with uveitis in the current study (2013–2015) was 4.6 years higher. A possible reason for the older age of patients with uveitis might be the rapid aging of the Japanese population [30]. Thus, it can be expected that the frequencies of herpetic iridocyclitis and intraocular lymphoma will be further increasing in the future in Japan. Indeed, the average age of patients with herpetic iridocyclitis or intraocular lymphoma in the current study was 62.5 ± 14.8 years and 72.0 ± 12.0 years, respectively. Further studies should be continued to clarify the trends of the patterns of uveitis.

The limitations of this study include its retrospective design, that it was conducted in a tertiary referral center, and that the study period was only 3 years. However, 750 patients were included in this study, and we believe that the current study’s findings could reflect the current trends of uveitis in Japan.

## Conclusions

The recent patterns of uveitis in the central Tokyo area revealed increasing trends of herpetic iridocyclitis and intraocular lymphoma. Because the patterns of uveitis are continuously changing, ongoing investigations of the predominant types of uveitis are needed.

## Abbreviations

AAU: Acute anterior uveitis
AZOOR: Acute zonal occult outer retinopathy
CMV: Cytomegalovirus
DNA: Deoxyribonucleic acid
HLA: Human leukocyte antigen
HSV: Herpes simplex virus
HTVV-1: Anti-human T-cell lymphotropic virus type I
Ig: Immunoglobulin
IgH: Immunoglobulin heavy chain
IL: Interleukin
PCR: Polymerase chain reaction
VKH: Vogt–Koyanagi–Harada disease
VZV: Varicella zoster virus

## References

[1] Durrani OM, Tehrani NN, Marr JE, Moradi P, Stavrou P, Murray PI (2004). Degree, duration, and causes of visual loss in uveitis. Br J Ophthalmol.

[2] Nakahara H, Kaburaki T, Takamoto M, Okinaga K, Matstuda J, Konno Y (2015). Statistical analyses of endogeneous uveitis patients (2007-2009) in Central Tokyo area and comparison with previous studies (1963-2006). Ocul Immunol Inflamm.

[3] Merrill PT, Kim J, Cox TA, Betorr CC, McCallum RM, Jaffe GJ (1997). Uveitis in the southeastern United States. Curr Eye Res.

[4] Goto H, Mochizuki M, Yamaki K, Kotake S, Usui M, Ohno S (2007). Epidemiological survey of intraocular inflammation in Japan. Jpn J Ophthalmol.

[5] Keino H, Nakashima C, Watanabe T, Taki W, Hayakawa R, Sugitani A (2009). Frequency and clinical features of intraocular inflammation in Tokyo. Clin Exp Ophthalmol.

[6] Al-Shakarchi FI (2014). Pattern of uveitis at a referral center in Iraq. Middle East Afr J Ophthalmol.

[7] Llorenç V, Mesquida M, Sainz de la Maza M, Keller J, Molins B, Espinosa G (2015). Epidemiology of uveitis in a western urban multiethnic population. Acta Ophthalmol.

[8] Baarsma GS (1992). The epidemiology and genetics of endogenous uveitis: a review. Curr Eye Res.

[9] Chen SC, Chuang CT, Chu MY, Sheu SJ (2017). Patterns and etiologies of uveitis at a tertiary referral center in Taiwan. Ocul Immunol Inflamm.

[10] Chou LC, Sheu SJ, Hong MC, Hsiao YC, Wu TT, Chuang CT (2003). Endogenous uveitis: experiences in Kaohsiung Veterans General Hospital. J Chin Med Assoc.

[11] Nakahara H, Kaburaki T, Tanaka R, Takamoto M, Ohtomo K, Karakawa A (2017). Frequency of uveitis in the central Tokyo area (2010–2012). Ocul Immunol Inflamm.

[12] Al Dhibi HA, Al Shamsi HA, Al-Mahmood AM, Al Taweel HM, Al Shamrani MA, Arevalo JF (2017). Patterns of uveitis in a tertiary care referral institute in Saudi Arabia. Ocul Immunol Inflamm.

[13] Ohguro N, Sonoda KH, Takeuchi M, Matsumura M, Mochizuki M (2012). The 2009 prospective multi-center epidemiologic survey of uveitis in Japan. Jpn J Ophthalmol.

[14] Jabs DA, Nussenblatt RB, Rosenbaum JT, Standardization of Uveitis Nomenclature (SUN) Working Group (2005). Standardization of uveitis nomenclature for reporting clinical data Results of the First International Workshop. Am J Ophthalmol.

[15] Suzuki Kurokawa M, Suzuki N (2004). Behcet’s disease. Clin Exp Med.

[16] The Japanese Society of Sarcoidosis and Other Granulomatous Disorders (2007). Diagnostic standard and guidelines for sarcoidosis-2006. Nippon Sarcoidosis Gakkai Zasshi.

[17] Kawaguchi T, Hanada A, Horie S, Sugamoto Y, Sugita S, Mochizuki M (2007). Evaluation of characteristic ocular signs and systemic investigations in ocular sarcoidosis patients. Jpn J Ophthalmol.

[18] Read RW, Holland GN, Rao NA, Tabbara KF, Ohno S, Arellanes-Garcia L (2001). Revised diagnostic criteria for Vogt-Koyanagi-Harada disease: report of an international committee on nomenclature. Am J Ophthalmol.

[19] Namba K, Goto H, Kaburaki T, Kitaichi N, Mizuki N, Asukata Y (2015). A major review: current aspects of ocular Behçet's disease in Japan. Ocul Immunol Inflamm.

[20] Ogawa M, Sugita S, Shimizu N, Watanabe K, Nakagawa I, Mochizuki M (2012). Broad-range real-time PCR assay for detection of bacterial DNA in ocular samples from infectious endophthalmitis. Jpn J Ophthalmol.

[21] Ogawa M, Sugita S, Watanabe K, Shimizu N, Mochizuki M (2012). Novel diagnosis of fungal endophthalmitis by broad-range real-time PCR detection of fungal 28S ribosomal DNA. Graefes Arch Clin Exp Ophthalmol.

[22] Cassoux N, Giron A, Bodaghi B, Tran TH, Baudet S, Davy F (2007). IL-10 measurement in aqueous humor for screening patients with suspicion of primary intraocular lymphoma. Invest Ophthalmol Vis Sci.

[23] Kaburaki T, Taoka K, Matsuda J, Yamashita H, Matsuda I, Tsuji H, et al. Combined intravitreal methotrexate and immunochemotherapy followed by reduced-dose whole-brain radiotherapy for newly diagnosed B-cell primary intraocular lymphoma. Br J Haematol. 2017; 10.1111/bjh.14848. 10.1111/bjh.14848 28699673

[24] Cimino L, Herbort CP, Aldigeri R, Salvarani C, Boiardi L (2009). Tuberculous uveitis, a resurgent and underdiagnosed disease. Int Ophthalmol.

[25] Oswal KS, Sivaraj RR, Murray PI, Stavrou P (2013). Clinical course and visual outcome in patients with diabetes mellitus and uveitis. BMC Res Notes.

[26] Mochizuki M, Tajima K, Watanabe T, Yamaguchi K (1994). Human T lymphotropic virus type 1 uveitis. Br J Ophthalmol.

[27] Francis PJ, Marinescu A, Fitzke FW, Bird AC, Holder GE (2005). Acute zonal occult outer retinopathy: towards a set of diagnostic criteria. Br J Ophthalmol.

[28] Quentin CD, Reiber H (2004). Fuchs heterochromic cyclitis: rubella virus antibodies and genome in aqueous humor. Am J Ophthalmol.

[29] Ohno S, Char DH, Kimura SJ, O'Connor GR (1977). HLA antigens and antinuclear antibody titres in juvenile chronic iridocyclitis. Br J Ophthalmol.

[30] Nishi N, Yoshizawa T, Okuda N (2017). Effects of rapid aging and lower participation rate among younger adults on the short-term trend of physical activity in the National Health and nutrition survey, Japan. Geriatr Gerontol Int.

[31] Rimland D, Moanna A (2010). Increasing incidence of herpes zoster among Veterans. Clin Infect Dis.

[32] Citterio G, Reni M, Gatta G, Ferreri AJM (2017). Primary central nervous system lymphoma. Crit Rev Oncol Hematol.

[33] Keino H, Watanabe T, Taki W, Nakayama M, Nakamura T, Yan K (2017). Clinical features of uveitis in children and adolescents at a tertiary referral Centre in Tokyo. Br J Ophthalmol.

